# Perspectives of primary care providers regarding multicancer early detection panels

**DOI:** 10.31744/einstein_journal/2024AO0771

**Published:** 2024-08-05

**Authors:** Benjamin E. Ueberroth, Richard J. Presutti, Alyssa McGary, Mitesh J. Borad, Neera Agrwal

**Affiliations:** 1 Department of Hematology/Oncology University of Colorado Anschutz School of Medicine Aurora CO United States Department of Hematology/Oncology, University of Colorado Anschutz School of Medicine, Aurora, CO, United States.; 2 Department of Family Medicine Mayo Clinic Jacksonville FL United States Department of Family Medicine, Mayo Clinic, Jacksonville, FL, United States.; 3 Department of Quantitative Health Sciences Mayo Clinic Scottsdale AZ United States Department of Quantitative Health Sciences, Mayo Clinic, Scottsdale, AZ, United States.; 4 Mayo Clinic Comprehensive Cancer Center Phoenix AZ United States Mayo Clinic Comprehensive Cancer Center, Phoenix, AZ, United States.; 5 Department of Medicine Mayo Clinic Phoenix AZ United States Department of Medicine, Mayo Clinic, Phoenix, AZ, United States.

**Keywords:** Early detection of cancer, Primary health care, High-value care, Delivery of health care, Quality of health care, Health equity, Surveys and questionnaires

## Abstract

**Objective:**

Multicancer early detection panels have recently become available to patients with healthcare provider prescriptions and available funds. These tests utilize circulating tumor DNA (ctDNA) to screen more than 50 cancers using a single blood sample. However, perspectives and data on how the deployment of these tests may impact the practices of primary care providers in terms of implementation, interpretation, documentation, and costs are limited. This study aimed to assess the perspectives of primary care providers regarding the integration of multicancer early detection panels into clinical practice.

**Methods:**

We used a survey to assess the opinions and perspectives of primary care providers, including physicians, nurse practitioners, and physician assistants, across a multistate, tertiary healthcare system. We used a single form consisting of novel questions on familiarity with multi-cancer early detection panels, cost, healthcare equity, documentation, medicolegal, and other concerns. The subgroup analysis was consistent with stratification based on familiarity with ctDNA-based tests and their roles in clinical practice.

**Results:**

Most respondents were unfamiliar with multicancer early detection panels and had not used ctDNA-based tests. Most primary care providers suggested that they would reorder multicancer early detection panel testing at 1- to 5-year intervals and prefer subspecialists for both ordering multicancer early detection panels as well as interpreting their results. Relative concerns differed between physicians and nonphysicians.

**Conclusion:**

The integration of multicancer early detection panels into primary care practice requires careful planning and consideration for the management of increased clinical load, interpretation of results, and cost management.

## Graphical abstract

**Figure:**
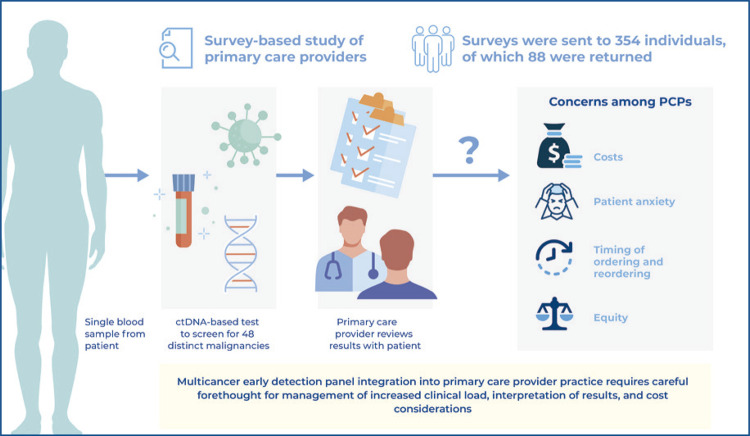

## INTRODUCTION

Early detection and diagnosis of cancer represent a booming industry, captivating research efforts and garnering considerable attention in clinical practice; this enhanced focus stems from the potential promise of these methods. One such endeavor that has recently gained increasing attraction is multicancer early detection panels (MCEDs). These panels encompass blood-based tests that are available in various forms and are capable of detecting several types of cancer across different stages; they are currently at different stages of development. Some utilize a single blood test, whereas others are being proposed as part of larger pipelines, including prespecified subsequent testing based on positive results.^(1,2)^ In general, these methods are based on the detection of circulating tumor DNA (ctDNA) and the assessment of various mutations present in spontaneously lysed tumor cells circulating within the blood.^(3)^ One such panel, the Galleri^®^ MCED (GRAIL, Inc, Menlo Park, CA, USA), is currently available to patients with a healthcare provider’s prescription through a laboratory-developed test (LDT) status from the Federal Drug Administration (FDA). The LDT status is a less rigorous process than the formal FDA review, wherein a test is only conducted at a single laboratory, as opposed to broad availability at numerous laboratories and/or health care centers as necessary for receiving FDA approval.^(4)^ The Galleri^®^ MCED is currently available for an out-of-pocket initial cost of $949 to the patient.^(5)^

The Galleri^®^ MCED detects genome-wide methylation changes, with the thresholds and specific changes determined through rigorous preclinical testing and data modeling.^(6)^ This single blood test can screen for the presence of more than 50 different cancers, both solid organ- and blood-based; some cancers have been reportedly detected at a significantly earlier stage than what is possible with the currently available screening methods.^(1)^ GRAIL as well as others have offered opinions on how these tests may alter clinical practice and patient experience, particularly within the primary care setting wherein they may first be offered.^(3,7-10)^ Significant effort has been devoted to cost modeling. Nonetheless, there will be significant learning curves in cost-effectiveness, a potential burden on clinical practice and clerical tasks, and patient anxiety regarding positive results.^(9,11)^ The scientific, clinical, and patient experience associated with this test are currently being investigated by the manufacturer GRAIL, the United Kingdom National Health Service, and others.^(12,13)^ The impact of the test on clinicians is uncertain, especially because there is direct marketing to consumers who may ask their primary care providers (PCPs) to order the test.

## OBJECTIVE

The aim of this study was to assess the current landscape of the perspectives of primary care providers regarding multicancer early detection panels, in particular the Galleri^®^, given its current availability to patients. As we continue to learn about these panels through real-world experience, it is imperative to consider the potential positive and negative aspects associated with the workflow, patient concerns, and clinician perspectives. This would allow all stakeholders to discuss these topics in the context of multicancer early detection panels.

## METHODS

This was a survey-based study of PCPs across the Mayo Clinic Enterprise, which includes three distinct yet interconnected physical locations (Rochester, MN; Phoenix, AZ; Jacksonville, FL, USA). This study was deemed “Exempt” by the Mayo Clinic Institutional Review Board. For the purposes of this study, physicians, nurse practitioners, and physician assistants within a department defined as “primary care” within the Mayo Clinic system were considered PCPs. The roles of “primary care” were confirmed with department chairs prior to serving to ensure a correct fit. Graduate medical trainees were excluded from the survey.

Based on the available literature, a custom was built to assess multiple potential MCED factors impacting both providers and patients (Survey 1). We assessed the overall familiarity with ctDNA and MCEDs, previous ordering and/or interpretation of ctDNA-based tests, estimation of insurance coverage and out-of-pocket costs to patients, estimated patient interest in such tests at current costs, interpretation of test performance (*e.g*., interpretation of reported sensitivity and specificity), preference for which provider specialty should be responsible for ordering these tests, frequency of ordering, age range for ordering, comfort level with interpretation of results and counseling patients and/or referral to subspeciality, pursuing subsequent evaluation and testing for positive results, potential burden of clinical time and/or documentation, medicolegal concerns, patient anxiety regarding positive results and/or false reassurance from negative result, and impact on adherence to age-appropriate cancer screening. The full survey questions and answers are presented in Table 1S, Supplementary Material.

After confirming the roles of PCPs, surveys were sent via email to an internal company. The study data were collected and managed using the REDCap electronic data capture tools hosted at the Mayo Clinic.^(14,15)^ No financial incentive or other support was offered for this survey. All results were collected anonymously. Questions were not mandatory; hence, an unanswered question is reported as “missing” in the dataset.

For analysis, participants were stratified based on “familiarity,” defined as follows: If a practitioner answered “First time hearing of them,” “Heard of them but Not Familiar with what they are,” or “Some degree of familiarity but have not ordered/interpreted,” then they were classified as “Not Familiar;” If they answered “Previously ordered/interpreted these tests” or “Routine/frequent use in everyday practice,” they were classified as “Familiar.” Similarly, an additional analysis was stratified into two groups based on the answer (“Yes/No”) to the question “Have you previously ordered other tests based on ctDNA?”. Finally, the participants were stratified for analysis as physicians or nurse practitioners/physician assistants (NPPAs).

Continuous variables were summarized using median and interquartile range (IQR), and categorical variables were summarized using the frequency and percentage. Kruskal-Wallis rank sum test was used for continuous variables, and Fisher’s Exact Test was used for categorical variables to assess the differences among the stratified groups. A p<0.05 was considered to be significant. Statistical software R 4.1.2 was used for analysis.

## RESULTS

**Figure 1:**
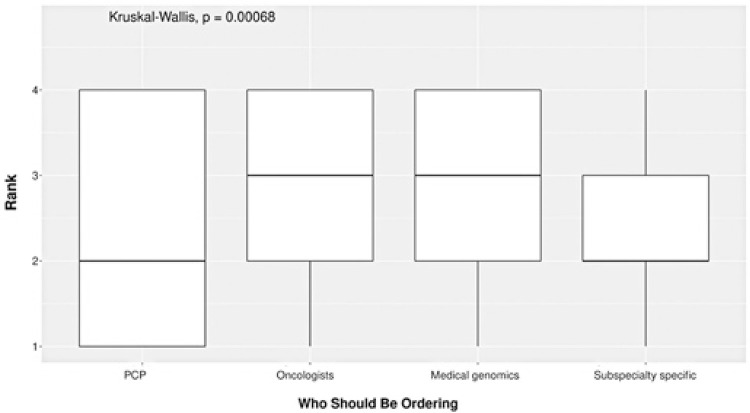
Results of Kruskal-Wallis rank sum test showing differences in preferences for “Who should be ordering?”

Surveys were sent to 354 individuals, and 88 of these were returned. A summary of particularly relevant and/or statistically significant questions is presented in table 1. The complete survey answers are tabulated in Table 1S to 4S, Supplemental Material. The majority of respondents were physicians (73%), with the remainder consisting of NPPAs. The majority of respondents were not familiar with MCEDs (82%) and had not previously ordered ctDNA-based testing of any type (87%). The majority indicated they would reorder this test at some interval (69%), with the predominant minority selecting a 5-year interval (37%). A semi-automated medical record feature was considered to be sufficient by the majority for reporting a negative result to a patient (83%); however, that was not the case for a positive MCED result (27%). The predominant minority chose to review a positive result during an in-person visit (42%), whereas a patient portal message was the most common choice for reporting a negative result to a patient (81%). A patient age range of 45-80 years at panel use was revealed based on the survey responses. Finally, a significant difference was noted among the responses to Q8 (“Who should be ordering…?”), with respondents preferring orders being placed by oncologists or medical genomics specialists as opposed to PCPs or subspeciality-specific providers (p<0.001; Figure 1).

**Table 1:** Summary of selected survey responses without stratification

Q1. Please indicate your role/position, n (%)	
Answer choice	
Physician assistant	3 (4)
Nurse practitioner	17 (23)
Physician	53 (73)
Missing	15
Q2. What is your level of familiarity with the Grail Galleri test and/or blood-based MCEDs in general?, n (%)	
First time hearing of them	35 (40)
Heard of them but not familiar with what they are	18 (21)
Some degree of familiarity but have not ordered/interpreted	18 (21)
Previously ordered/interpreted these tests	14 (16)
Routine/frequent use in everyday practice	2 (2)
Missing	1
Q3. Have you previously ordered other test(s) based on ctDNA?, n (%)	
No	75 (87)
Yes	11 (13)
Missing	2
Q4. For an interested patient with an initially negative GRAIL Galleri MCED result, how often would you re-order this test?, n (%)	
Every year (annually)	16 (21)
Every 5 years	28 (37)
Every 10 years	8 (11)
Once only; would not order again	13 (17)
Choose not to answer	10 (13)
Missing	13
Q5a. A semi-automated medical record feature would be sufficient medicolegal documentation for a POSITIVE MCED result, n (%)	
Agree	20 (27)
Disagree	54 (73)
Missing	14
Q5b. A semi-automated medical record feature would be sufficient medicolegal documentation for a NEGATIVE MCED result, n (%)	
Agree	62 (83)
Disagree	13 (17)
Missing	13
Q6a. How would you review a POSITIVE MCED test result with a patient in most cases?, n (%)	
Patient portal/electronic communication	5 (7)
Phone call	27 (37)
In-person visit	31 (42)
Send to subspecialist for interpretation	7 (10)
Choose not to answer	3 (4)
Missing	15
Q6b. How would you review a NEGATIVE MCED test result with a patient in most cases?, n (%)	
Patient portal/electronic communication	59 (81)
Phone call	5 (7)
In-person visit	3 (4)
Send to subspecialist for interpretation	2 (3)
Choose not to answer	4 (5)
Missing	15
Q7. What is the age range for which you would order this test?	
Age in which provider would order MCED	Median, years (IQR)
Youngest (would not order in patients below this age)	45 (30-50)
Oldest (would not order in patients above this age)	80 (75-80)
Q8. Who should be ordering the GRAIL Galleri test? (1=Least appropriate, 4=Most appropriate)	
Answer choice/rank	Median (IQR*)*
Primary care providers	2.0 (1.0-4.0)
Oncologists	3.0 (2.0-4.0)
Medical genomics specialists	3.0 (2.0-4.0)
Subspeciality-specific providers (*e.g*., GI provider orders for GI malignancy screen)	2.0 (2.0-3.0)
Kruskal-Wallis rank sum test (Figure 1)	p<0.001

MCED: multicancer early detection panel; ctDNA: circulating tumor DNA; GI: gastroenterologist.

**Figure 2:**
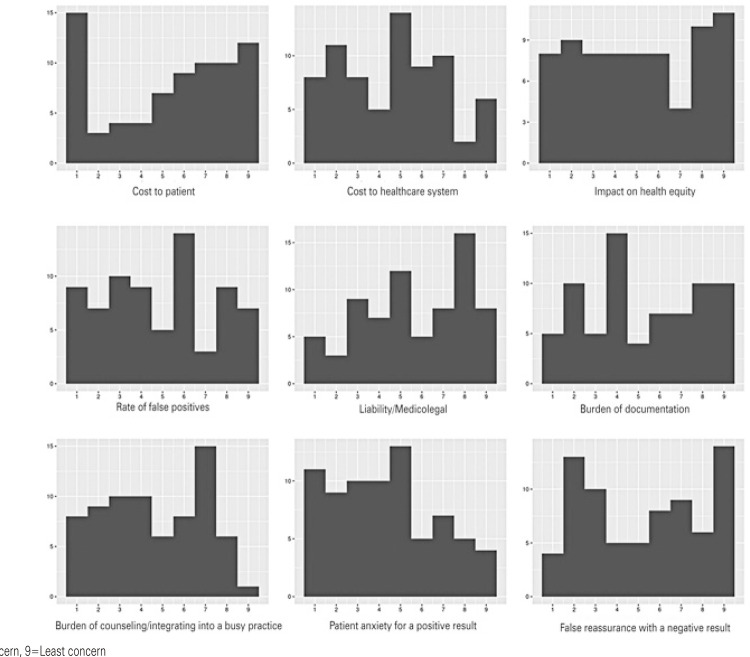
Histograms showing concerns regarding the use of multicancer early detection panels in primary care practice

A matrix of nine potential concerns was developed and queried in the survey. Each concern was ranked discretely from 1-9, with 1 being the most concerning and 9 the least concerning. The results of this matrix are tabulated below (Table 2), and the distributions are shown (Figure 2). Liability and cost-to-patient concerns were ranked relatively high, whereas perceived time burden and patient anxiety were ranked relatively lower.

**Table 2:** Potential concerns related to the use of multicancer early detection panels in primary care practice

Potential concern	Median (IQR)
Liability/medicolegal	6.0 (4.0-8.0)
Cost to patient	6.0 (3.0-8.0)
False reassurance with a negative test	5.5 (3.0-8.0)
Burden of documentation	5.0 (3.0-8.0)
Impact on health equity (*i.e*., access to a $979 test)	5.0 (3.0-8.0)
Rate of false positives	5.0 (3.0-7.0)
Cost to healthcare system (*e.g*., downstream testing, referrals)	5.0 (2.0-6.0)
Burden of counseling/integrating into a busy practice	4.0 (3.0-7.0)
Patient anxiety with a positive result	4.0 (2.0-6.0)

X-axis: Rating of concern; 1=Greatest concern, 9=Least concern

Y-axis: Number of responses.

When participants were stratified by “familiarity” as defined above (Table 3), multiple significant differences were noted. Unsurprisingly, those familiar with MCEDs were significantly more likely to have ordered other ctDNA tests (p<0.001), whereas those unfamiliar with MCEDs were significantly more likely to overestimate the potential insurance coverage (p<0.001) and estimated that fewer patients would be interested in undergoing this test at the current cost (p=0.032). Furthermore, those familiar with MCEDs were more likely to estimate that they would reorder the test within a shorter interval (p=0.030), favoring annual reordering (54% *versus* 17%). Overall, there was reduced confidence in the unfamiliar group with regard to interpreting either result (negative or positive); interpreting a positive result reached a significantly lower confidence level in the unfamiliar group than in the familiar group (p=0.013). Those familiar with the test were significantly more likely to order disease-directed subsequent evaluations over subspeciality referral than those unfamiliar with MCEDs (p=0.008).

**Table 3:** Selected responses stratified by familiarity with multicancer early detection panels

Question & answer choices	Familiar (n=16) n (%)	Not familiar (n=71) n (%)	p value
Q1. Have you previously ordered tests based on ctDNA?			<0.001
No	9 (56)	66 (94)	
Yes	7 (44)	4 (6)	
Missing	0	1	
Q2. On average, to what extent will health insurance cover the cost of GRAIL Galleri multicancer early detection panel?			<0.001
Fully covered (*i.e*., no cost to patient)	0 (0)	1 (2)	
Partially covered (*i.e*., copay)	0 (0)	5 (9)	
Conditionally covered (*e.g.*, certain ages)	1 (7)	30 (54)	
Not covered (*i.e*., 100% out-of-pocket)	13 (93)	20 (36)	
Missing	2	15	
Q3. What percentage of your patients would be interested in undergoing this test at its current price ($949)?			0.032
Very few, if any (<20%)	4 (31)	34 (54)	
Some but not many (<50%)	5 (38)	26 (41)	
Many but not all (>50%)	3 (23)	2 (3)	
All or nearly all (>80%)	1 (8)	1 (2	
Missing	3	8	
Q4. For an interested patient with an initially negative MCED^†^ result, how often would you re-order this test?			0.030
Every year (annually)	7 (54)	9 (17)	
Every 5 years	3 (23)	25 (48)	
Every 10 years	0 (0)	8 (15)	
Once only, would not order again	3 (23)	10 (19)	
Missing	3	19	
Q5a. Would you feel comfortable interpreting a NEGATIVE MCED result with a patient?			0.058
No	0 (0)	16 (26)	
Yes	13 (0)	46 (74)	
Missing	3	9	
Q5b. Would you feel comfortable interpreting a POSITIVE MCED result with a patient?			0.013
No	2 (15)	34 (55)	
Yes	11 (85)	28 (45)	
Missing	3	9	
Q6. For a positive MCED result, what would be your next step?			0.008
Order disease-directed evaluation	12 (92)	27 (46)	
Refer to subspecialist and defer further testing to the subspeciality consultant	1 (8)	17 (29)	
Refer to subspecialist and concurrently order disease-directed evaluation	0 (0)	15 (25)	

ctDNA: circulating tumor DNA; MCED: multicancer early detection panel.

When participants were stratified based on the previous ordering of other ctDNA tests, only one question, in addition to familiarity with these tests, demonstrated a significant difference in responses. The participants who had prior experience in working with ctDNA were less likely to be interested in undergoing MCED testing at the current cost than those without prior ctDNA experience ones (p=0.043).

Finally, when participants were stratified based on their roles (NPPA or physician) (Table 4), physicians were significantly more likely to express familiarity with MCEDs than NPPAs (p=0.039). NPPAs estimated some degree of insurance coverage compared to physicians, 57% of NPPAs compared to 36% of physicians estimated conditional coverage (*e.g*., covered for certain age groups, with certain insurance plans), and 60% of physicians compared to 29% of NPPAs expected no insurance coverage and 100% out-of-pocket cost to patients. NPPAs were significantly more likely to report MCEDs as effective at detecting most early-stage cancers (65% *versus* 29%, p=0.019) and identifying subspecialists (not PCPs) as the ideal interpreters of positive results (together with patients) than the physicians (p=0.004). There was a significant difference in the distribution of concerns regarding the time spent counseling patients undergoing MCED testing (p=0.040) and interpreting/communicating results to patients (p=0.017). Physicians were significantly more likely to order a subsequent disease-directed evaluation for a positive result than NPPAs, who favored referral to subspeciality provider(s) with or without concurrent disease-directed evaluation (p=0.007). The youngest age at which physicians would order MCED testing was significantly higher than that preferred by NPPAs (median 50 *versus* 30 years, p=0.004), who were more likely to consider testing patients at a significantly older age (80 *versus* 75 years, p=0.026). When ranking relative potential concerns regarding MCED testing, physicians reported the rate of false positives as a significantly greater concern than NPPAs did (p=0.014), whereas the burden of counseling/time (p=0.021) and burden of documentation (p=0.05) were significantly lower concerns for physicians than for NPPAs.

**Table 4:** Responses for selected questions stratified by role (Nurse practitioner or physician assistant or physician)

Questions & answer choices			NPPA (n=20) n (%)			Physician (n=53) n (%)	p value
Q1. What is your level of familiarity with the GRAIL Galleri test and/or MCEDs in general?							
First time hearing of them			12 (60)			14 (26)	0.039
Heard of them but not familiar			4 (20)			13 (25)	
Some familiarity but not previously used			4 (20)			13 (25)	
Previously ordered/interpreted MCED			0 (0)			11 (21)	
Routine/frequent use in practice			0 (0)			2 (4)	
Q2. On average, to what extent will health insurance cover the cost of GRAIL Galleri multicancer early detection panel?							
Fully covered (*i.e*., no cost to patient)			1 (7)			0 (0)	0.061
Partially covered (*e.g*., copay)			1 (7)			2 (4)	
Conditionally covered (*i.e*., age groups)			8 (57)			17 (36)	
Not covered (*i.e*., 100% out-of-pocket)			4 (29)			28 (60)	
Missing			6			6	
Q3. This test is effective at detecting most early-stage cancers							
Yes			11 (65)			15 (29)	0.019
No			6 (35)			36 (71)	
Missing			3			2	
Q4. In your opinion, who should interpret the results of an MCED test to the patient?							
Primary care providers			1 (6)			20 (42)	0.004
Oncologists			6 (33)			4 (8)	
Medical genomics			8 (44%)			13 (27%)	
Subspeciality based on MCED result			3 (17%)			11 (23%)	
Missing			2			5	
Q5. How concerned are you about the amount of time you anticipate spending on…							
Counseling patients on whether to undergo MCED testing?							
Not at all			0 (0)			8 (15)	0.040
A little			6 (32)			9 (17)	
Somewhat			7 (37)			13 (25)	
Quite			1 (5)			15 (28)	
Very			5 (26)			8 (15)	
Interpreting the results of MCEDs and communicating results to patients?							
Not at all			0 (0)			7 (13)	0.017
A little			2 (11)			7 (13)	
Somewhat			10 (53)			11 (21)	
Quite			1 (5)			16 (30)	
Very			6 (32)			12 (23)	
Q6. For a positive MCED result, what would be your next step?							
Order disease-directed evaluation			5 (25)			33 (65)	0.007
Refer to a subspecialist and defer further testing to the subspeciality consultant			9 (45)			9 (18)	
Refer to a subspecialist and concurrently order disease-directed evaluation			6 (30)			9 (18)	
Missing			0			2	
Q7. What is the age range for which you would order the GRAIL Galleri MCED test?							
Age	NPPA-median, years (IQR)			Physician-median, years (IQR)			
Youngest	30.0 (22.0-42.5)			50 (40.0-50.0)			0.004
Oldest	75.0 (73.8-80.0)			80.0 (75.0 -80.0)			0.026
Q8. Rank your concerns regarding MCEDs (1=Greatest concern, 9=Least concern)							
Concern		NPPA-median (IQR)			Physician-median (IQR)		
Rate of false positives		6.0 (4.8-8.0)			4.0 (2.0-6.0)		0.014
Burden of counseling/time		3.5 (2.0-4.2)			6.0 (3.0-7.0)		0.021
Burden of documentation		3.5 (2.0-7.0)			5.0 (4.0-8.0)		0.050
Patient anxiety (positive result)		3.0 (1.8-5.5)			4.0 (3.0-6.0)		0.359
Cost to patient		6.5 (4.0-8.0)			6.0 (2.0-8.0)		0.362
False reassurance (negative result)		7.0 (3.0-8.2)			5.0 (3.0-7.0)		0.469
Cost to healthcare system		5.0 (3.0-6.0)			5.0 (2.0-7.0)		0.604
Liability/Medicolegal		5.5 (4.0-7.0)			6.0 (3.0-8.0)		0.726
Impact on health equity		5.5 (3.8-7.0)			5.0 (3.0-8.0)		0.985

NPPA: nurse practitioner or physician assistant; MCED: multicancer early detection panel.

## DISCUSSION

Overall, the majority of respondents indicated their role as physicians (73%), with nearly a quarter being NPs (23%) and the remaining PAs (4%). A predominant minority (47%) worked in the family medicine department (patients of all ages), with the remainder split across adult medicine primary care departments. Regarding cost considerations, nearly half of the respondents expected at least some degree of insurance coverage (44%) despite the current status where the entire expense is faced by patients. Once aware of the price (949 USD), 87% of the respondents felt that less than half of their patients would be interested in undergoing this test at the current price. This presents a significant opportunity for provider education and is not necessarily a concern specific to MCEDs but testing in general. Any educational and/or advertising materials being presented to PCPs who may order MCEDs would ideally highlight the out-of-pocket cost for the patient and the exact cost of the procedure; thus, all PCPs who may order these tests can appropriately relay the costs to patients when discussing the costs and benefits before proceeding. Although not specific to MCEDs, cost awareness at the time of ordering has been associated with reduced testing and, thereby, costs.^(16)^ There is also precedent where a computer-based decision-support tool can directly lead to healthcare savings in primary care; perhaps, that could be considered with MCEDs.^(17)^

Regarding costs, the intertwined nature of medicine, politics, and private industry is remarkably complicated and affects MCED adoption. Other researchers have also shared concerns and opinions on cost ramifications.^(7,18)^ Although there is some momentum for Medicare coverage of MCEDs as screening tests,^(19)^ there is no current coverage regarding downstream testing for a positive MCED result, which is likely to be more significant than the MCED costs themselves. In addition to the perspectives cited previously which express these concerns, the National Cancer Institute states, “There is little known about whether the cost of a diagnostic workup for a positive MCD result would be covered by insurance.”^(20)^ Currently, it is incumbent upon healthcare professionals to not only address the initial expenses but also the potential for a significant financial burden that patients may face as a consequence of pursuing disease-specific further testing following a positive MCED result.

For all respondents, the median age range for ordering MCEDs (45-80 years) was largely in line with that utilized in studies leading to MCED implementation.^(1,7)^ Furthermore, this is a logical age group epidemiologically, given that most age-appropriate cancer screenings (other than cervical cancer) are carried out across either this exact age range or within a few years.^(21)^ Interestingly, NPPA respondents were more likely to consider testing at a younger age (median 30 *versus* 50 years, p=0.004), whereas physicians were more likely to consider MCED testing at an older age (median 80 *versus* 75 years, p=0.026). Given that age is a primary (and unmodifiable) “risk factor” for malignancy, the older age limit is likely more malleable; however, the younger age limit certainly merits further discussion. With the overall cancer risk beginning to increase in the 40-50-year age range and peaking in the 60-70-year age range, one should consider if testing below 50 years, and even more so below 40 years of age, may open up MCEDs to significantly increased false positives and lower yields.^(22)^ This being said, a primary aim of using MCEDs is “early detection,” in other words earlier stage cancers, and unsurprisingly, when cancer is detected, earlier stage cancers are generally associated with younger age.^(23,24)^ The earlier ordering considered in NPPA group is also congruent with NPPAs answering “yes” significantly more than physicians to the question regarding MCED performance in detecting early-stage cancers (Table 4, Q3; p=0.019). Perhaps including specifics regarding relative performance in detection by stage in MCED educational materials would be useful here, potentially limiting the testing of younger patients, which is associated with uncertain benefits. Overall, this reinforces the need for an individualized approach to ordering these tests, extensive risk/benefit discussions, and significant caution when ordering MCEDs for relatively younger patients, given that primary studies have largely focused on the >50-year age group.^(1,6,11)^

Primary care providers generally felt comfortable managing negative results, primarily through semi-automated documentation; however, result management requires careful handling. Emerging areas of study could include artificial intelligence, which may be especially worth considering for the review of negative MCED results based on the comfort of PCPs with negative results in this study.^(25,26)^ Approximately half of the respondents felt comfortable ordering disease-directed evaluations for a positive MCED result (with or without concurrent subspeciality referral), and there was a significantly higher proportion of those who felt comfortable independently ordering this testing in the group familiar with MCEDs. To effectively manage MCED results, particularly in cases of positive findings, the establishment of well-defined protocols is crucial. Such protocols will facilitate accurate prediction of the impact on the workload of a given practice as the utilization of MCEDs escalates. This could potentially free up more time for PCPs or the subspecialists that see the majority of these patients, allowing for more focused patient care. At the institution where this study was conducted, PCPs were the only groups currently ordering these tests, and it is currently unclear how positive results, in general or with specific tumors/organs, are handled. Oncologists are generally the other group most commonly aware of these tests, followed by medical genomics specialists, whereas subspeciality providers are sometimes unaware that MCEDs are already available. Direct perspectives of patients regarding whom they might desire as result interpreters, among all of these topics, would be of great utility and merit further study.

Across all participants, medicolegal concerns (liability) and costs to patients were considered the more relevant concerns than the others listed (Table 2). The burden of counseling and patient anxiety with positive results were considered relatively low-level concerns by the respondents. When stratified by familiarity with MCEDs, the rate of false positives was higher for those unfamiliar with MCEDs, although the difference was not statistically significant (p=0.054). This is congruent with some of the other results discussed above, in that those unfamiliar with MCEDs are understandably less familiar with the test characteristics (sensitivity, specificity, positive predictive value, and negative predictive value). This group and others have previously offered insights into the interpretation of test characteristics, which may represent useful educational materials for those unfamiliar with MCEDs.^(9,10)^The most interesting results derived from the relative concern ranking were the different concerns between NPPAs and physicians. Nurse practitioners/physician assistants indicated a significantly greater concern for the time spent counseling about undergoing MCED testing (p=0.021) and documentation (p=0.050), whereas physicians showed a significantly greater concern for the rate of false-positive MCED results (p=0.014).

Positive results require a clear and thoughtful approach to limit the time burden and uncertainty. One could consider a “prepackaged” approach, with any positivity such as a subsequent PET-CT for any positive MCED. This has been tangentially studied with a different MCED placed in a pipeline, including PET-CT. Although the yield was low, it may be more applicable in this context; nevertheless, this warrants further investigation.^(27)^Alternatively, order sets specific to the MCED signal of origin include colonoscopy for a colon cancer MCED signal, mammography for breast cancer, and bone marrow biopsy for blood-based cancers. If these are to be considered, it would be prudent to perform prospective studies on such pipelines to determine the potential harm as well as the costs of subsequent testing. Nevertheless, it is evident that positive MCED results should be followed by further investigations.

Certain questions with “negative” findings in this survey also warrant discussion. Across the entire group and consistent with stratifications, it was clear that PCPs do not feel that this approach replaces age-appropriate cancer screening (*e.g*., colorectal, cervical, lung, and breast cancer), which is in line with the advice of Galleri.^(1)^ The messaging regarding this has been clear, and it is key to educate patients to avoid the concern of false reassurance from negative MCED results. As discussed above, a negative MCED result seemed to be less concerning for PCP in terms of interpreting, time spent discussing, and/or time spent documenting. Furthermore, managing a negative MCED result seems feasibly within the scope of any primary care practice based on our data. Specifically, from a PCP perspective, a patient portal message, potentially a semi-automated one, seems sufficient for informing patients of negative MCED results.

The ramifications of health equity merit further discussion. It is interesting to gauge the current media, socio-political, and layperson perspectives on equity, with frequent mentions of these tests to improve health equity gaps.^(5,19,20,28,29)^While that certainly may be the case, those discussions all hinge at minimum on broad insurance coverage of MCED tests and subsequent downstream testing for a positive result. While the former may have some promise in the bills that have been introduced to the US Congress regarding Medicare coverage, the latter is completely devoid of discussion at this point and raises many, if not the majority, of cost-related concerns. Currently, at an out-of-pocket expense of $949 to patients, these tests will skew toward more affluent individuals who are already likely to have relatively easy access to healthcare, thus making it unlikely to improve cancer diagnosis disparities. For insurance coverage to materialize, particularly in an optimal scenario where both MCED and any subsequent testing are fully covered, it would necessitate not just the involvement of Medicare but also a broader engagement from more widely available insurance carriers, such as Medicaid and those found on the health insurance marketplace in the United States. Notably, the National Cancer Institute is on record reporting that “More research is needed to understand whether MCD tests improve access or worsen healthcare disparities.”^(20)^

Nevertheless, this study has several limitations. It represents a single multistate healthcare system with a large tertiary referral practice. Extrapolation to smaller practices, especially those with larger proportions of underserved and lower-socioeconomic status patients, may be limited. The survey response rate was approximately 25%; therefore, the majority of surveys went unanswered, although the distribution of primary care departments was relatively even. Private practice and specific practice setups (*e.g*., concierge medicine) may also contribute to varied responses. Survey-based studies certainly have limitations, such as estimating the feelings of respondents when they are not actually present in a given situation (*i.e*., hypothetical), recall bias, and the influence of one survey question or information on subsequent questions. Further studies directly assessing the opinions of patients should supplement the opinions of care providers with the preferences of their patients. The sample size was relatively small, a characteristic often observed in single-center studies. This aspect is further accentuated when conducting stratified analyses of small subgroups. Finally, questions pertaining to workflow, especially regarding those who should order these tests, may be subject to confounders, such as practices already burdened by high patient volumes that are not necessarily directly concerned with MCEDs themselves, but more so, are an addition to an already busy clinical practice.

## CONCLUSION

Multicancer early detection panels provide a novel method for screening multiple cancers using a single blood sample. The test performance varies significantly across cancer types, cancer stages, and patient-specific factors. Who orders these tests and, more importantly, who is responsible for the review of positive results and downstream testing requires significant forethought when integrating these tests into everyday primary care practice. EHR-based solutions may help mitigate the documentation burden, and clearly defined protocols regarding referrals for positive results, in particular, may help mitigate the potential increase in time burden for primary care providers.

## SUPPLEMENTARY MATERIAL

Perspectives of primary care providers regarding multicancer early detection panels


