# Supplementary material for: Perspectives of primary care providers regarding multicancer early detection panels
Source: Einstein (Sao Paulo). 2024 Aug 5;22:eAO0771. doi: 10.31744/einstein_journal/2024AO0771 (PMC11323832; doi:10.31744/einstein_journal/2024AO0771)
Supplement: Supplementary file 1 [file 2317-6385-eins-22-eAO0771-suppl01.pdf]

SUPPLEMENTARY MATERIAL

Perspectives of primary care providers regarding multicancer early detection panels

Benjamin E. Ueberroth, Richard J. Presutti, Alyssa McGary, Mitesh J. Borad, Neera Agrwal

DOI: 10.31744/einstein_journal/2024AO0771

Table 1S. Overall summary of the results

Familiarity and costs	Overall (n=88)
What is your level of familiarity with the GRAIL Galleri test and/or blood-based multicancer early detection panels (MCEDs) in general?, n (%)	
First time hearing of them	35 (40)
Heard of them but not familiar with what they are	18 (21)
Some degree of familiarity but have not ordered/interpreted	18 (21)
Previously ordered/interpreted these tests	14 (16)
Routine/frequent use in everyday practice	2 (2)
Missing	1
Have you previously ordered other test(s) based on circulating tumor DNA (ctDNA)?, n (%)	
No	75 (87)
Yes	11 (13)
Missing	2
On average, to what extent will health insurance cover the cost of GRAIL Galleri multicancer early detection panel?, n (%)	
Fully covered (*i.e.*, no cost to patient)	1 (1)
Partially covered (*i.e.*, copay or similar)	5 (6)
Conditionally covered (*i.e.*, certain ages, groups, and insurance packages)	31 (37)
Not covered (100% out-of-pocket cost to patient)	33 (40)
Choose not to answer	13 (16)
Missing	5
What is your best estimate of the out-of-pocket cost of the GRAIL test for most patients?, n (%)	
$100	9 (11)
$1000	43 (52)
$1500	20 (24)
$10000	3 (4)
Choose not to answer	8 (10)
Missing	5
What percentage of YOUR patients would be interested in undergoing this test at its current price ($949)?, n (%)	
Very few, if any (<20%)	38 (48)
Some but not many (<50%)	31 (39)
Many but not all (>50%)	5 (6)
All or nearly all (>80%)	2 (2)
Choose not to answer	4 (5)
Missing	8
**Test characteristics and performance**	**Overall (n=88)**
This test is effective at detecting most early-stage cancers, n (%)	
Yes	28 (36)
No	44 (56)
Choose not to answer	6 (8)
Missing	10

continue...

...Continuation

Test characteristics and performance	Overall (n=88)
This test is effective at detecting most early-stage cancers, n (%)	
This test is an effective replacement for current screening (*e.g.*, Pap smear, colonoscopy, mammography, low-dose lung CT, and prostate-specific antigen), n (%)	
Yes	1 (1)
No	72 (92)
Choose not to answer	5 (6)
Missing	10
**The GRAIL-Galleri test should be ordered (1 = Least appropriate, 4 = Most appropriate)**	**Overall (n=88)**
Primary care providers (PCPs)	
Missing	11
Mean (SD)	2.3 (1.3)
Median (IQR)	2.0 (1.0–4.0)
Range	1.0 - 4.0
Oncologists	
Missing	11
Mean (SD)	2.6 (1.1)
Median (IQR)	3.0 (2.0–4.0)
Range	1.0 - 4.0
Medical genomics	
Missing	11
Mean (SD)	2.9 (1.1)
Median (IQR)	3.0 (2.0–4.0)
Range	1.0-4.0
Subspeciality specific (*i.e.*, GI providers order to screen for GI cancers and OBGYNs for gynecologic cancers)	
Missing	11
Mean (SD)	2.2 (0.8)
Median (IQR)	2.0 (2.0–3.0)
Range	1.0-4.0
**Ordering the test**	**Overall (n=88)**
For an interested patient with an initially negative Galleri GRAIL MCED result, how often would you reorder this test?, n (%)	
Every year (annually)	16 (21)
Every 5 years	28 (37)
Every 10 years	8 (11)
Once only; would not order again	13 (17)
Choose not to answer	10 (13)
Missing	13
What is the YOUNGEST age for which you would order a GRAIL Galleri MCED test? (please enter age in years)	
Missing	13
Mean (SD)	40.8 (13.9)
Median (IQR)	45.0 (30.0–50.0)
Range	12.0-99.0
What is the OLDEST age for which you would order the GRAIL Galleri MCED test? (please enter age in years)	
Missing	13
Mean (SD)	77.5 (8.8)
Median (IQR)	80.0 (75.0–80.0)
Range	24.0-99.0

continue...

...Continuation

Interpreting the test	Overall (n=88)
Would you feel comfortable interpreting a NEGATIVE MCED result with a patient? (Negative = no cancer detected), n (%)	
No	16 (21)
Yes	59 (79)
Missing	13
Would you feel comfortable interpreting a POSITIVE MCED result with a patient? (Positive = cancer detected), n (%)	
No	36 (48)
Yes	39 (52)
Missing	13
In your opinion, who should interpret the results of a GRAIL Galleri MCED test with the patient (regardless of who ordered the test)?, n (%)	
Primary care providers (PCPs)	21 (28)
Oncologists	10 (13)
Medical genomics specialists	22 (29)
Subspeciality based on signal of origin (*e.g.*, GI provider for GI signal and OBGYN for gynecologic signal), n (%)	15 (20)
Choose not to answer	7 (9)
Missing	13
**Concerns and documentation**	**Overall (n=88)**
How concerned are you about the amount of time you anticipate spending on counseling patients on whether to undergo the GRAIL Galleri test?, n (%)	
Not at all	9 (12)
A little	15 (20)
Somewhat	20 (27)
Quite	17 (23)
Very	13 (17)
Choose not to answer	1 (1)
Missing	13
How concerned are you about the amount of time you anticipate spending on interpreting the results of GRAIL Galleri tests and communicating results to patients?, n (%)	
Not at all	8 (11)
A little	9 (12)
Somewhat	22 (29)
Quite	17 (23)
Very	18 (24)
Choose not to answer	1 (1)
Missing	13
A semi-automated electronic medical record feature (*e.g.*, Epic Smartphrase) would be sufficient medicolegal documentation for a POSITIVE result, n (%)	
Agree	20 (27)
Disagree	54 (73)
Missing	14
A semi-automated electronic medical record feature (*e.g.*, Epic Smartphrase) would be sufficient medicolegal documentation for a NEGATIVE result, n(%)	
Agree	62 (83)
Disagree	13 (17)
Missing	13
**Next steps for a positive test**	**Overall (n=88)**
For a positive MCED result, what would be your next step?, n (%)	
Order disease-directed evaluation (*e.g.*, EGD for esophageal MCED signal)	39 (53)
Refer for oncology and/or medical genomics consultation and defer further testing to the subspeciality consultant	18 (24)
Refer to oncology and/or medical genomics specialists and concurrently order disease-directed evaluation	15 (20)
Choose not to answer	2 (3)
Missing	14

continue...

...Continuation

Next steps for a positive test	Overall (n=88)
How comfortable would you feel with ordering disease-directed subsequent testing for a positive GRAIL Galleri MCED test?, n (%)	
Not at all	6 (8)
Comfortable for a limited subset of cancers	29 (39)
Comfortable doing this with most/all cancers	36 (49)
Choose not to answer	3 (4)
Missing	14
**Concerns regarding the Galleri GRAIL MCED test (1 = Greatest concern, 9 = Least concern)**	**Overall (n=88)**
Cost to patient	
Missing	14
Mean (SD)	5.4 (2.9)
Median (IQR)	6.0 (3.0, 8.0)
Range	1.0 - 9.0
Cost to healthcare system (*e.g.*, increase in downstream testing and subspeciality referral)	
Missing	15
Mean (SD)	4.6 (2.4)
Median (IQR)	5.0 (2.0, 6.0)
Range	1.0 - 9.0
Impact on health equity (*i.e.*, access to a $979 test)	
Missing	14
Mean (SD)	5.1 (2.7)
Median (IQR)	5.0 (3.0, 8.0)
Range	1.0 - 9.0
Rate of false positives	
Missing	15
Mean (SD)	4.8 (2.6)
Median (IQR)	5.0 (3.0, 7.0)
Range	1.0 - 9.0
Liability/Medicolegal	
Missing	15
Mean (SD)	5.6 (2.4)
Median (IQR)	6.0 (4.0, 8.0)
Range	1.0 - 9.0
Burden of documentation	
Missing	15
Mean (SD)	5.2 (2.6)
Median (IQR)	5.0 (3.0, 8.0)
Range	1.0 - 9.0
Burden of counseling/integrating into a busy practice	
Missing	15
Mean (SD)	4.6 (2.3)
Median (IQR)	4.0 (3.0, 7.0)
Range	1.0 - 9.0
Patient anxiety for a positive result	
Missing	14
Mean (SD)	4.3 (2.4)
Median (IQR)	4.0 (2.0, 6.0)
Range	1.0 - 9.0
False reassurance with a negative result	
Missing	14
Mean (SD)	5.3 (2.7)
Median (IQR)	5.5 (3.0, 8.0)
Range	1.0 - 9.0

continue...

...Continuation

Reviewing the test	Overall (n=88)
How would you review a POSITIVE Galleri GRAIL test result with a patient in most cases?	
Patient portal/electronic communication	5 (7)
Phone call	27 (37)
In-person visit	31 (42)
Send to subspecialist for interpretation	7 (10)
Choose not to answer	3 (4)
Missing	15
How you review a NEGATIVE Galleri GRAIL with a patient in most cases?, n (%)	
Patient portal/electronic communication	59 (81)
Phone call	5 (7)
In-person visit	3 (4)
Send to subspecialist for interpretation	2 (3)
Choose not to answer	4 (5)
Missing	15
**Department and role**	**Overall (n=88)**
Please indicate the department in which you primarily see patients, n (%)	
Community Internal Medicine (CIM)	17 (23)
Family Medicine (FAM)	34 (47)
General Internal Medicine (GIM; including Executive Health, Development, Consultative Medicine, and International Health)	21 (29)
Medallion (MDL)	0 (0)
Women's Health Internal Medicine (WHIM)	0 (0)
None of the above	1 (1)
Missing	15
Please indicate your role/position	
Physician Assistant	3 (4)
Nurse Practitioner	17 (23)
Physician	53 (73)
None of the above	0 (0)
Missing	15

Table 2S. Summary of familiarity with multicancer early detection panels and the associated costs

Familiarity with MCEDs	Familiar (n=16)	Not Familiar (n=71)	p value
Have you previously ordered other test(s) based on circulating tumor DNA (ctDNA)?, n (%)			<0.001*
No	9 (56)	66 (94)	
Yes	7 (44)	4 (6)	
Missing	0	1	
On average, to what extent will health insurance cover the cost of GRAIL Galleri multicancer early detection panel?, n (%)			<0.001*
Fully covered (*i.e.*, no cost to patient)	0 (0)	1 (2)	
Partially covered (*i.e.*, copay or similar)	0 (0)	5 (9)	
Conditionally covered (*i.e.*, certain ages, groups, and insurance packages)	1 (7)	30 (54)	
Not covered (100% out-of-pocket cost to patient)	13 (93)	20 (36)	
Missing	2	15	
What is your best estimate of the out-of-pocket cost of the GRAIL test for most patients?, n (%)			0.304*
$100	0 (0)	9 (15)	
$1000	11 (79)	32 (52)	
$1500	3 (21)	17 (28)	
$10000	0 (0)	3 (5)	
Missing	2	10	
What percentage of YOUR patients would be interested in undergoing this test at its current price ($949)?			0.032*
Very few, if any (<20%)	4 (31)	34 (54)	
Some but not many (<50%)	5 (38)	26 (41)	
Many but not all (>50%)	3 (23)	2 (3)	
All or nearly all (>80%)	1 (8)	1 (2)	
Missing	3	8	
**Test characteristics and performance**	**Familiarity with MCEDs**		**p value**
	**Familiar (n=16)**	**Not Familiar (n=71)**	
This test is effective at detecting most early-stage cancers, n (%)			0.754*
Yes	4 (31)	24 (41)	
No	9 (69)	35 (59)	
Missing	3	12	
This test is an effective replacement for current screening (*e.g.*, Pap smear, colonoscopy, mammography, low-dose lung CT, and prostate-specific antigen), n (%)			1.000*
Yes	0 (0)	1 (2)	
No	13 (100)	59 (98)	
Missing	3	11	
**Who should be ordering the GRAIL Galleri test (1 = Least appropriate, 4 = Most appropriate)**	**Familiarity with MCEDs**		**p value**
	**Familiar (n=16)**	**Not Familiar (n=71)**	
Primary care providers (PCPs)			0.326†
Missing	3	7	
Mean (SD)	2.7 (1.4)	2.2 (1.3)	
Median (IQR)	3.0 (1.0, 4.0)	2.0 (1.0, 4.0)	
Range	1.0 - 4.0	1.0 - 4.0	
Oncologists			0.961†
Missing	3	7	
Mean (SD)	2.6 (1.2)	2.6 (1.1)	
Median (IQR)	3.0 (2.0, 4.0)	3.0 (2.0, 4.0)	
Range	1.0 - 4.0	1.0 - 4.0	
Medical genomics			0.235†
Missing	3	7	
Mean (SD)	2.5 (1.1)	2.9 (1.1)	
Median (IQR)	3.0 (2.0, 3.0)	3.0 (2.0, 4.0)	
Range	1.0 - 4.0	1.0 - 4.0	
Subspeciality specific (*i.e.*, GI providers order to screen for GI cancers and OBGYNs for gynecologic cancers)			0.936†
Missing	3	7	
Mean (SD)	2.2 (0.7)	2.2 (0.8)	
Median (IQR)	2.0 (2.0, 3.0)	2.0 (2.0, 3.0)	
Range	1.0 - 3.0	1.0 - 4.0	

continue...

...Continuation

Ordering the test	Familiarity with MCEDs		p value
	Familiar (n=16)	Not Familiar (n=71)	
For an interested patient with an initially negative Galleri GRAIL MCED result, how often would you reorder this test?, n (%)			0.030*
Every year (annually)	7 (54)	9 (17)	
Every 5 years	3 (23)	25 (48)	
Every 10 years	0 (0)	8 (15)	
Once only; would not order again	3 (23)	10 (19)	
Missing	3	19	
What is the YOUNGEST age for which you would order a GRAIL Galleri MCED test? (please enter age in years)			0.663†
Missing	3	9	
Mean (SD)	42.5 (10.8)	40.4 (14.5)	
Median (IQR)	45.0 (40.0, 50.0)	45.0 (30.0, 50.0)	
Range	21.0–55.0	12.0–99.0	
What is the OLDEST age for which you would order the GRAIL Galleri MCED test? (please enter age in years)			0.254†
Missing	3	9	
Mean (SD)	79.9 (6.5)	77.0 (9.1)	
Median (IQR)	80.0 (75.0, 85.0)	79.5 (75.0, 80.0)	
Range	70.0–90.0	24.0–99.0	

Interpreting the test	Familiarity with MCEDs		p value
	Familiar (n=16)	Not Familiar (n=71)	
Would you feel comfortable interpreting a NEGATIVE MCED result with a patient? (Negative = no cancer detected), n (%)			0.058*
No	0 (0)	16 (26)	
Yes	13 (100)	46 (74)	
Missing	3	9	
Would you feel comfortable interpreting a POSITIVE MCED result with a patient? (Positive = cancer detected), n (%)			0.013*
No	2 (15)	34 (55)	
Yes	11 (85)	28 (45)	
Missing	3	9	
In your opinion, who should interpret the results of a GRAIL Galleri MCED test with the patient (regardless of who ordered the test)?, n (%)			0.210*
Primary care providers (PCPs)	7 (58)	14 (25)	
Oncologists	1 (8)	9 (16)	
Medical genomics specialists	2 (17)	20 (36)	
Subspeciality based on signal of origin (*e.g.*, GI provider for GI signal and OBGYN for gynecologic signal)	2 (17)	13 (23)	
Missing	4	15	

Concerns and documentation	Familiarity with MCEDs		p value
	Familiar (n=16)	Not Familiar (n=71)	
How concerned are you about the amount of time you anticipate spending on counseling patients on whether to undergo the GRAIL Galleri test?, n (%)			0.530*
Not at all	2 (15)	7 (11)	
A little	2 (15)	13 (21)	
Somewhat	5 (38)	15 (25)	
Quite	1 (8)	16 (26)	
Very	3 (23)	10 (16)	
Missing	3	10	
How concerned are you about the amount of time you anticipate spending on interpreting the results of GRAIL Galleri tests and communicating results to patients?, n (%)			0.362*
Not at all	2 (15)	6 (10)	
A little	3 (23)	6 (10)	
Somewhat	2 (15)	20 (33)	
Quite	4 (31)	13 (21)	
Very	2 (15)	16 (26)	
Missing	3	10	

continue...

...Continuation

Concerns and documentation	Familiarity with MCEDs		p value
	Familiar (n=16)	Not Familiar (n=71)	
A semi-automated electronic medical record feature (*e.g.*, Epic smartphrase) would be sufficient medicolegal documentation for a POSITIVE result, n (%)			0.739*
Agree	4 (31)	16 (26)	
Disagree	9 (69)	45 (74)	
Missing	3	10	
A semi-automated electronic medical record feature (*e.g.*, Epic smartphrase) would be sufficient medicolegal documentation for a NEGATIVE result, n (%)			0.108*
Agree	13 (100)	49 (79)	
Disagree	0 (0)	13 (21)	
Missing	3	9	

Next steps for a positive test	Familiarity with MCEDs		p value
	Familiar (n=16)	Not Familiar (n=71)	
For a positive MCED result, what would be your next step?, n (%)			0.008*
Order disease-directed evaluation (*e.g.*, EGD for esophageal MCED signal)	12 (92)	27 (46)	
Refer for oncology and/or medical genomics consultation and defer further testing to the subspeciality consultant	1 (8)	17 (29)	
Refer to oncology and/or medical genomics specialists and concurrently order disease-directed evaluation	0 (0)	15 (25)	
Missing	3	12	
How comfortable would you feel with ordering disease-directed subsequent testing for a positive GRAIL Galleri MCED test?, n (%)			0.328*
Not at all	0 (0)	6 (10)	
Comfortable for a limited subset of cancers	4 (31)	25 (43)	
Comfortable doing this with most/all cancers	9 (69)	27 (47)	
Missing	3	13	

Concerns regarding the Galleri GRAIL MCED test (1 = Greatest concern, 9 = Least concern)	Familiarity with MCEDs		p value
	Familiar (n=16)	Not Familiar (n=71)	
Cost to patient			0.397†
Missing	3	10	
Mean (SD)	6.0 (2.8)	5.2 (2.9)	
Median (IQR)	6.0 (6.0, 8.0)	6.0 (2.0, 8.0)	
Range	1.0-9.0	1.0-9.0	
Cost to healthcare system (*e.g.*, increase in downstream testing and subspeciality referral)			0.556†
Missing	3	11	
Mean (SD)	5.0 (2.1)	4.5 (2.5)	
Median (IQR)	5.0 (4.0, 7.0)	5.0 (2.0, 6.0)	
Range	2.0-9.0	1.0-9.0	
Impact on health equity (*i.e.*, access to a $979 test)			0.274†
Missing	3	10	
Mean (SD)	5.8 (3.4)	4.9 (2.5)	
Median (IQR)	8.0 (3.0, 9.0)	5.0 (3.0, 7.0)	
Range	1.0-9.0	1.0-9.0	
Rate of false positives			0.054†
Missing	3	11	
Mean (SD)	3.6 (2.6)	5.1 (2.5)	
Median (IQR)	4.0 (1.0, 5.0)	5.5 (3.0, 7.0)	
Range	1.0-8.0	1.0-9.0	
Liability/Medicolegal			0.896†
Missing	3	11	
Mean (SD)	5.6 (2.5)	5.7 (2.4)	
Median (IQR)	7.0 (3.0, 8.0)	5.5 (4.0, 8.0)	
Range	1.0-8.0	1.0-9.0	
Burden of documentation			0.861†
Missing	3	11	
Mean (SD)	5.1 (2.0)	5.2 (2.7)	
Median (IQR)	4.0 (4.0, 6.0)	5.0 (3.0, 8.0)	
Range	2.0-9.0	1.0-9.0	

continue...

...Continuation

Concerns regarding the Galleri GRAIL MCED test (1 = Greatest concern, 9 = Least concern)	Familiarity with MCEDs		p value
	Familiar (n=16)	Not Familiar (n=71)	
Burden of counseling/integrating into a busy practice			0.754†
Missing	3	11	
Mean (SD)	4.5 (2.1)	4.6 (2.4)	
Median (IQR)	4.0 (3.0, 6.0)	4.5 (2.8, 7.0)	
Range	1.0–7.0	1.0–9.0	
Patient anxiety for a positive result			0.448†
Missing	3	10	
Mean (SD)	3.8 (2.5)	4.4 (2.4)	
Median (IQR)	3.0 (2.0, 5.0)	4.0 (3.0, 6.0)	
Range	1.0–8.0	1.0–9.0	
False reassurance with a negative result			0.736†
Missing	3	10	
Mean (SD)	5.5 (2.7)	5.2 (2.8)	
Median (IQR)	6.0 (3.0, 7.0)	5.0 (3.0, 8.0)	
Range	2.0–9.0	1.0–9.0	
**Reviewing the test**	**Familiarity with MCEDs**		**p value**
	**Familiar (n=16)**	**Not Familiar (n=71)**	
How would you review a POSITIVE Galleri GRAIL test result with a patient in most cases?, n (%)			0.956*
Patient portal/electronic communication	1 (8)	4 (7)	
Phone call	6 (46)	21 (37)	
In-person visit	5 (38)	26 (46)	
Send to subspecialist for interpretation	1 (8)	6 (11)	
Missing	3	14	
How you review a NEGATIVE Galleri GRAIL with a patient in most cases?, n (%)			0.841*
Patient portal/electronic communication	12 (100)	47 (82)	
Phone call	0 (0)	5 (9)	
In-person visit	0 (0)	3 (5)	
Send to subspecialist for interpretation	0 (0)	2 (4)	
Missing	4	14	
**Department and role**	**Familiarity with MCEDs**		**p value**
	**Familiar (n=16)**	**Not Familiar (n=71)**	
Please indicate the department in which you primarily see patients, n (%)			
Community Internal Medicine (CIM)	0 (0)	17 (28)	
Family Medicine (FAM)	0 (0)	34 (57)	
General Internal Medicine (GIM; including Executive Health, Development, Consultative Medicine, and International Health)	13 (100)	8 (13)	
None of the above	0 (0)	1 (2)	
Missing	3	11	
Please indicate your role/position			
Physician Assistant	0 (0)	3 (5)	
Nurse Practitioner	0 (0)	17 (28)	
Physician	13 (100)	40 (67)	
Missing	3	11	

*Fisher's Exact Test; †Kruskal-Wallis rank sum test.

Table 3S. Summary of previous ordering of other test(s) based on circulating tumor DNA

Previously ordered other test(s) based on ctDNA?	Familiarity with MCEDs		p value
	No (n=75)	Yes (n=11)	
What is your level of familiarity with the GRAIL Galleri test and/or blood-based multicancer early detection panels (MCEDs) in general?, n (%)			<0.001*
First time hearing of them	31 (41)	3 (27)	
Heard of them but not familiar with what they are	18 (24)	0 (0)	
Some degree of familiarity but have not ordered/interpreted	17 (23)	1 (9)	
Previously ordered/interpreted these tests	9 (12)	5 (45)	
Routine/frequent use in everyday practice	0 (0)	2 (18)	
On average, to what extent will health insurance cover the cost of GRAIL Galleri multicancer early detection panel?, n (%)			0.553*
Fully covered (*i.e.*, no cost to patient)	1 (2)	0 (0)	
Partially covered (*i.e.*, copay or similar)	4 (7)	1 (10)	
Conditionally covered (*i.e.*, certain ages, groups, insurance packages)	28 (47)	3 (30)	
Not covered (100% out-of-pocket cost to patient)	27 (45)	6 (60)	
Missing	15	1	
What is your best estimate of the out-of-pocket cost of the GRAIL test for most patients?, n (%)			0.089*
$100	8 (12)	1 (10)	
$1000	35 (54)	8 (80)	
$1500	20 (31)	0 (0)	
$10000	2 (3)	1 (10)	
Missing	10	1	
What percentage of YOUR patients would be interested in undergoing this test at its current price ($949)?, n (%)			0.043*
Very few, if any (<20%)	36 (54)	2 (22)	
Some but not many (<50%)	27 (40)	4 (44)	
Many but not all (>50%)	3 (4)	2 (22)	
All or nearly all (>80%)	1 (1)	1 (11)	
Missing	8	2	
**Test characteristics and performance**	**Test characteristics and performance**		**p value**
	**No (n=75)**	**Yes (n=11)**	
This test is effective at detecting most early-stage cancers, n (%)			0.703*
Yes	24 (38)	4 (50)	
No	40 (62)	4 (50)	
Missing	11	3	
This test is an effective replacement for current screening (*e.g.*, Pap smear, colonoscopy, mammography, low-dose lung CT, and prostate-specific antigen), n (%)			0.110*
Yes	0 (0)	1 (12)	
No	65 (100)	7 (88)	
Missing	10	3	
**Who should be ordering the GRAIL Galleri test (1 = Least appropriate, 4 = Most appropriate)**	**Previously ordered other test(s) based on ctDNA?**		**p value**
	**No (n=75)**	**Yes (n=11)**	
Primary care providers (PCPs)			0.338†
Missing	6	3	
Mean (SD)	2.3 (1.3)	2.8 (1.3)	
Median (IQR)	2.0 (1.0, 4.0)	3.0 (1.8, 4.0)	
Range	1.0-4.0	1.0-4.0	
Oncologists			0.463†
Missing	6	3	
Mean (SD)	2.6 (1.1)	2.9 (1.4)	
Median (IQR)	3.0 (2.0, 3.0)	3.5 (1.8, 4.0)	
Range	1.0-4.0	1.0-4.0	
Medical genomics			0.163†
Missing	6	3	
Mean (SD)	2.9 (1.1)	2.4 (1.1)	
Median (IQR)	3.0 (2.0, 4.0)	2.5 (1.8, 3.0)	
Range	1.0-4.0	1.0-4.0	

continue...

...Continuation

Who should be ordering the GRAIL Galleri test (1 = Least appropriate, 4 = Most appropriate)	Previously ordered other test(s) based on ctDNA? No (n=75)	Yes (n=11)	p value
Subspeciality specific (*i.e.*, GI providers order to screen for GI cancers and OBGYNs for gynecologic cancers)			0.520†
Missing	6	3	
Mean (SD)	2.2 (0.8)	2.0 (0.8)	
Median (IQR)	2.0 (2.0, 3.0)	2.0 (1.8, 2.2)	
Range	1.0–4.0	1.0–3.0	
**Ordering the test**	**Previously ordered other test(s) based on ctDNA? No (n=75)**	**Yes (n=11)**	**p value**
For an interested patient with an initially negative Galleri GRAIL MCED result, how often would you re-order this test?, n (%)			0.070*
Every year (annually)	12 (20)	4 (67)	
Every 5 years	27 (46)	1 (17)	
Every 10 years	7 (12)	1 (17)	
Once only; would not order again	13 (22)	0 (0)	
Missing	16	5	
What is the YOUNGEST age for which you would order a GRAIL Galleri MCED test? (please enter age in years)			0.558†
Missing	7	4	
Mean (SD)	40.7 (13.9)	41.4 (15.0)	
Median (IQR)	45.0 (30.0, 50.0)	50.0 (33.5, 50.0)	
Range	12.0–99.0	18.0–55.0	
What is the OLDEST age for which you would order the GRAIL Galleri MCED test? (please enter age in years)			0.209†
Missing	7	4	
Mean (SD)	77.2 (9.0)	80.7 (5.3)	
Median (IQR)	79.5 (75.0, 80.0)	80.0 (77.5, 82.5)	
Range	24.0–99.0	75.0–90.0	
**Interpreting the test**	**Previously ordered other test(s) based on ctDNA? No (n=75)**	**Yes (n=11)**	**p value**
Would you feel comfortable interpreting a NEGATIVE MCED result with a patient? (Negative = no cancer detected), n (%)			0.334*
No	16 (24)	0 (0)	
Yes	52 (76)	7 (100)	
Missing	7	4	
Would you feel comfortable interpreting a POSITIVE MCED result with a patient? (Positive = cancer detected), n (%)			0.109*
No	35 (51)	1 (14)	
Yes	33 (49)	6 (86)	
Missing	7	4	
In your opinion, who should interpret the results of a GRAIL Galleri MCED test with the patient (regardless of who ordered the test)?, n (%)			0.423*
Primary care providers (PCPs)	18 (30)	3 (43)	
Oncologists	8 (13)	2 (29)	
Medical genomics specialists	21 (34)	1 (14)	
Subspeciality based on signal of origin (e.g., GI provider for GI signal and OBGYN for gynecologic signal)	14 (23)	1 (14)	
Missing	14	4	
**Concerns and documentation**	**Previously ordered other test(s) based on ctDNA? No (n=75)**	**Yes (n=11)**	**p value**
How concerned are you about the amount of time you anticipate spending on counseling patients on whether to undergo the GRAIL Galleri test?, n (%)			0.362*
Not at all	7 (10)	2 (29)	
A little	13 (19)	2 (29)	
Somewhat	18 (27)	2 (29)	
Quite	17 (25)	0 (0)	
Very	12 (18)	1 (14)	
Missing	8	4	
How concerned are you about the amount of time you anticipate spending on interpreting the results of GRAIL Galleri tests and communicating results to patients?, n (%)			0.067*
Not at all	6 (9)	2 (29)	
A little	7 (10)	2 (29)	
Somewhat	22 (33)	0 (0)	
Quite	15 (22)	2 (29)	
Very	17 (25)	1 (14)	
Missing	8	4	

continue...

...Continuation

Concerns and documentation	Previously ordered other test(s) based on ctDNA?		p value
	No (n=75)	Yes (n=11)	
A semi-automated electronic medical record feature (*e.g.*, Epic smartphrase) would be sufficient medicolegal documentation for a POSITIVE result, n (%)			1.000*
Agree	18 (27)	2 (29)	
Disagree	49 (73)	5 (71)	
Missing	8	4	
A semi-automated electronic medical record feature (*e.g.*, Epic smartphrase) would be sufficient medicolegal documentation for a NEGATIVE result, n (%)			1.000*
Agree	56 (82)	6 (86)	
Disagree	12 (18)	1 (14)	
Missing	7	4	
**Next steps for a positive test**	**Previously ordered other test(s) based on ctDNA?**		**p value**
	**No (n=75)**	**Yes (n=11)**	
For a positive MCED result, what would be your next step?, n (%)			0.872*
Order disease-directed evaluation (*e.g.*, EGD for esophageal MCED signal)	34 (52)	5 (71)	
Refer for oncology and/or medical genomics consultation and defer further testing to the subspeciality consultant	17 (26)	1 (14)	
Refer to oncology and/or medical genomics specialists and concurrently order disease-directed evaluation	14 (22)	1 (14)	
Missing	10	4	
How comfortable would you feel with ordering disease-directed subsequent testing for a positive GRAIL Galleri MCED test?, n (%)			0.593*
Not at all	6 (9)	0 (0)	
Comfortable for a limited subset of cancers	27 (42)	2 (29)	
Comfortable doing this with most/all cancers	31 (48)	5 (71)	
Missing	11	4	
**Concerns regarding the Galleri-GRAIL MCED test (1 = Greatest concern, 9 = Least concern)**	**Previously ordered other test(s) based on ctDNA?**		**p value**
	**No (n=75)**	**Yes (n=11)**	
Cost to patient			0.337†
Missing	8	4	
Mean (SD)	5.2 (2.9)	6.4 (2.2)	
Median (IQR)	6.0 (2.0, 8.0)	7.0 (5.0, 8.0)	
Range	1.0–9.0	3.0–9.0	
Cost to healthcare system (*e.g.*, increase in downstream testing and subspeciality referral)			0.887†
Missing	9	4	
Mean (SD)	4.7 (2.5)	4.4 (2.0)	
Median (IQR)	5.0 (2.0, 6.0)	4.0 (3.0, 6.0)	
Range	1.0–9.0	2.0–7.0	
Impact on health equity (*i.e.*, access to a $979 test)			0.260†
Missing	8	4	
Mean (SD)	5.0 (2.6)	6.1 (3.7)	
Median (IQR)	5.0 (3.0, 7.5)	9.0 (3.0, 9.0)	
Range	1.0–9.0	1.0–9.0	
Rate of false positives			0.664†
Missing	9	4	
Mean (SD)	4.9 (2.6)	4.4 (2.6)	
Median (IQR)	5.0 (3.0, 7.0)	5.0 (2.5, 6.0)	
Range	1.0–9.0	1.0–8.0	
Liability/Medicolegal			0.917†
Missing	9	4	
Mean (SD)	5.6 (2.4)	5.7 (2.8)	
Median (IQR)	5.5 (4.0, 8.0)	7.0 (4.0, 8.0)	
Range	1.0–9.0	1.0–8.0	
Burden of documentation			0.461†
Missing	9	4	
Mean (SD)	5.2 (2.7)	5.9 (1.8)	
Median (IQR)	4.5 (3.0, 8.0)	6.0 (4.5, 6.5)	
Range	1.0–9.0	4.0–9.0	

continue...

...Continuation

Concerns regarding the Galleri-GRAIL MCED test (1 = Greatest concern, 9 = Least concern)	Previously ordered other test(s) based on ctDNA?		p value
	No (n=75)	Yes (n=11)	
Burden of counseling/integrating into a busy practice			0.438†
Missing	9	4	
Mean (SD)	4.7 (2.3)	4.0 (2.4)	
Median (IQR)	4.5 (3.0, 7.0)	4.0 (2.0, 6.0)	
Range	1.0–9.0	1.0–7.0	
Patient anxiety for a positive result			0.280†
Missing	8	4	
Mean (SD)	4.4 (2.4)	3.4 (2.3)	
Median (IQR)	4.0 (3.0, 6.0)	2.0 (2.0, 4.0)	
Range	1.0–9.0	2.0–8.0	
False reassurance with a negative result			0.467†
Missing	8	4	
Mean (SD)	5.3 (2.7)	4.6 (2.8)	
Median (IQR)	6.0 (3.0, 8.0)	5.0 (2.5, 6.0)	
Range	1.0–9.0	1.0–9.0	
**Reviewing the test**	**Previously ordered other test(s) based on ctDNA?**		**p value**
	**No (n=75)**	**Yes (n=11)**	
How would you review a POSITIVE Galleri GRAIL test result with a patient in most cases?, n (%)			1.000*
Patient portal/electronic communication	5 (8)	0 (0)	
Phone call	24 (38)	3 (43)	
In-person visit	27 (43)	4 (57)	
Send to a subspecialist for interpretation	7 (11)	0 (0)	
Missing	12	4	
How you review a NEGATIVE Galleri GRAIL with a patient in most cases?, n (%)			0.624*
Patient portal/electronic communication	54 (86)	5 (83)	
Phone call	4 (6)	1 (17)	
In-person visit	3 (5)	0 (0)	
Send to subspecialist for interpretation	2 (3)	0 (0)	
Missing	12	5	
**Department and role**	**Previously ordered other test(s) based on ctDNA?**		**p value**
	**No (n=75)**	**Yes (n=11)**	
Please indicate the department in which you primarily see patients, n (%)			
Community Internal Medicine (CIM)	17 (26)	0 (0%)	
Family Medicine (FAM)	32 (48)	2 (29)	
General Internal Medicine (GIM; including Executive Health, Development, Consultative Medicine, and International Health)	16 (24)	5 (71)	
None of the above	1 (2)	0 (0)	
Missing	9	4	
Please indicate your role/position			
Physician Assistant	3 (5)	0 (0)	
Nurse Practitioner	16 (24)	1 (14)	
Physician	47 (71)	6 (86)	
Missing	9	4	

*Fisher's Exact Test; †Kruskal-Wallis rank sum test.

Table 4S. Comparison of the results for physicians *versus* non-physicians

Role/Physician	Familiarity and cost		p value
	Not physician (n=20)	Physician (n=53)	
What is your level of familiarity with the GRAIL Galleri test and/or blood-based multicancer early detection panels (MCEDs) in general?, n (%)			0.039*
First time hearing of them	12 (60)	14 (26)	
Heard of them but not familiar with what they are	4 (20)	13 (25)	
Some degree of familiarity but have not ordered/interpreted	4 (20)	13 (25)	
Previously ordered/interpreted these tests	0 (0)	11 (21)	
Routine/frequent use in everyday practice	0 (0)	2 (4)	
Have you previously ordered other test(s) based on circulating tumor DNA (ctDNA)?, n (%)			0.665*
No	19 (95)	47 (89)	
Yes	1 (5)	6 (11)	
On average, to what extent will health insurance cover the cost of GRAIL Galleri multicancer early detection panel?, n (%)			0.061*
Fully covered (*i.e.*, no cost to patient)	1 (7)	0 (0)	
Partially covered (*i.e.*, copay or similar)	1 (7)	2 (4)	
Conditionally covered (*i.e.*, certain ages, groups, and insurance packages)	8 (57)	17 (36)	
Not covered (100% out-of-pocket cost to patient)	4 (29)	28 (60)	
Missing	6	6	
What is your best estimate of the out-of-pocket cost of the GRAIL test for most patients?, n (%)			0.112*
$100	3 (19)	4 (8)	
$1000	6 (38)	34 (68)	
$1500	6 (38)	11 (22)	
$10000	1 (6)	1 (2)	
Missing	4	3	
What percentage of YOUR patients would be interested in undergoing this test at its current price ($949)?, n (%)			1.000*
Very few, if any (<20%)	9 (53)	27 (52)	
Some but not many (<50%)	7 (41)	20 (38)	
Many but not all (>50%)	1 (6)	4 (8)	
All or nearly all (>80%)	0 (0)	1 (2)	
Missing	3	1	
**Fisher's Exact Test for Count Data<br>Test characteristics and performance**	**Role/Physician**		**p value**
	**Not physician (n=20)**	**Physician (n=53)**	
This test is effective at detecting most early-stage cancers, n (%)			0.019*
Yes	11 (65)	15 (29)	
No	6 (35)	36 (71)	
Missing	3	2	
This test is an effective replacement for current screening (*e.g.*, Pap smear, colonoscopy, mammography, low-dose lung CT, and prostate-specific antigen), n (%)			1.000*
Yes	0 (0)	1 (2)	
No	18 (100)	50 (98)	
Missing	2	2	
**Who should be ordering the GRAIL Galleri test (1 = Least appropriate, 4 = Most appropriate)**	**Role/Physician**		**p value**
	**Not physician (n=20)**	**Physician (n=53)**	
Primary care providers (PCPs)			0.519†
Mean (SD)	2.1 (1.3)	2.4 (1.3)	
Median (IQR)	1.5 (1.0, 3.2)	2.0 (1.0, 4.0)	
Range	1.0 - 4.0	1.0 - 4.0	
Oncologists			0.868†
Mean (SD)	2.5 (1.2)	2.6 (1.1)	
Median (IQR)	2.5 (1.8, 4.0)	3.0 (2.0, 3.0)	
Range	1.0 - 4.0	1.0 - 4.0	
Medical genomics			0.780†
Mean (SD)	2.9 (1.1)	2.9 (1.1)	
Median (IQR)	3.0 (2.0, 4.0)	3.0 (2.0, 4.0)	
Range	1.0 - 4.0	1.0 - 4.0	
Subspeciality specific (i.e., GI providers order to screen for GI cancers and OBGYNs for gynecologic cancers)			0.173†
Mean (SD)	2.5 (0.8)	2.1 (0.8)	
Median (IQR)	2.0 (2.0, 3.0)	2.0 (2.0, 3.0)	
Range	1.0 - 4.0	1.0 - 4.0	

continue...

...Continuation

Ordering the test	Role/Physician		p value
	Not physician (n=20)	Physician (n=53)	
For an interested patient with an initially negative Galleri GRAIL MCED result, how often would you re-order this test?, n (%)			0.184*
Every year (annually)	4 (24)	12 (26)	
Every 5 years	8 (47)	18 (39)	
Every 10 years	4 (24)	4 (9)	
Once only; would not order again	1 (6)	12 (26)	
Missing	3	7	
What is the YOUNGEST age for which you would order a GRAIL Galleri MCED test? (please enter age in years)			0.004†
Mean (SD)	32.2 (12.8)	43.6 (13.2)	
Median (IQR)	30.0 (22.0, 42.5)	50.0 (40.0, 50.0)	
Range	12.0 - 50.0	18.0 - 99.0	
What is the OLDEST age for which you would order the GRAIL Galleri MCED test? (please enter age in years)			0.026†
Mean (SD)	73.4 (13.1)	79.0 (6.1)	
Median (IQR)	75.0 (73.8, 80.0)	80.0 (75.0, 80.0)	
Range	24.0 - 90.0	65.0 - 99.0	
**Interpreting the test**	**Role/Physician**		**p value**
	**Not physician (n=20)**	**Physician (n=53)**	
Would you feel comfortable interpreting a NEGATIVE MCED result with a patient? (Negative = no cancer detected), n (%)			1.000*
No	4 (20)	12 (23)	
Yes	16 (80)	41 (77)	
Would you feel comfortable interpreting a POSITIVE MCED result with a patient? (Positive = cancer detected), n (%)			0.121*
No	13 (65)	23 (43)	
Yes	7 (35)	30 (57)	
In your opinion, who should interpret the results of a GRAIL Galleri MCED test with the patient (regardless of who ordered the test)?, n (%)			0.004*
Primary care providers (PCPs)	1 (6)	20 (42)	
Oncologists	6 (33)	4 (8)	
Medical genomics specialists	8 (44)	13 (27)	
Subspeciality based on signal of origin (*e.g.*, GI provider for GI signal and OBGYN for gynecologic signal)	3 (17)	11 (23)	
Missing	2	5	
**Concerns and documentation**	**Role/Physician**		**p value**
	**Not physician (n=20)**	**Physician (n=53)**	
How concerned are you about the amount of time you anticipate spending on counseling patients on whether to undergo the GRAIL Galleri test?, n (%)			0.040*
Not at all	0 (0)	8 (15)	
A little	6 (32)	9 (17)	
Somewhat	7 (37)	13 (25)	
Quite	1 (5)	15 (28)	
Very	5 (26)	8 (15)	
Missing	1	0	
How concerned are you about the amount of time you anticipate spending on interpreting the results of GRAIL Galleri tests and communicating results to patients?, n (%)			0.017*
Not at all	0 (0)	7 (13)	
A little	2 (11)	7 (13)	
Somewhat	10 (53)	11 (21)	
Quite	1 (5)	16 (30)	
Very	6 (32)	12 (23)	
Missing	1	0	
A semi-automated electronic medical record feature (*e.g.*, Epic smartphrase) would be sufficient medicolegal documentation for a POSITIVE result, n (%)			1.000*
Agree	5 (25)	14 (26)	
Disagree	15 (75)	39 (74)	
A semi-automated electronic medical record feature (*e.g.*, Epic smartphrase) would be sufficient medicolegal documentation for a NEGATIVE result, n (%)			1.000*
Agree	17 (85)	43 (81)	
Disagree	3 (15)	10 (19)	

continue...

...Continuation

Fisher's Exact Test for Count Data<br>Next steps for a positive test	Role/Physician		p value
	Not physician (n=20)	Physician (n=53)	
For a positive MCED result, what would be your next step?, n (%)			0.007*
Order disease-directed evaluation (*e.g.*, EGD for esophageal MCED signal)	5 (25)	33 (65)	
Refer for oncology and/or medical genomics consultation and defer further testing to the subspeciality consultant	9 (45)	9 (18)	
Refer to oncology and/or medical genomics and concurrently order disease-directed evaluation	6 (30)	9 (18)	
Missing	0	2	
How comfortable would you feel with ordering disease-directed subsequent testing for a positive GRAIL Galleri MCED test?, n (%)			0.101*
Not at all	4 (21)	2 (4)	
Comfortable for a limited subset of cancers	7 (37)	22 (43)	
Comfortable doing this with most/all cancers	8 (42)	27 (53)	
Missing	1	2	
**Concerns regarding the GRAIL-Galleri MCED test (1 = Greatest concern, 9 = Least concern).**	**Role/Physician**		**p value**
	**Not physician (n=20)**	**Physician (n=53)**	
Cost to patient			0.362†
Mean (SD)	5.8 (2.8)	5.1 (2.9)	
Median (IQR)	6.5 (4.0, 8.0)	6.0 (2.0, 8.0)	
Range	1.0 - 9.0	1.0 - 9.0	
Cost to healthcare system (*e.g.*, increase in downstream testing and subspeciality referral)			0.604†
Mean (SD)	4.9 (2.2)	4.5 (2.5)	
Median (IQR)	5.0 (3.0, 6.0)	5.0 (2.0, 7.0)	
Range	1.0 - 9.0	1.0 - 9.0	
Impact on health equity (*i.e.*, access to a $979 test)			0.985†
Mean (SD)	5.1 (2.4)	5.1 (2.9)	
Median (IQR)	5.5 (3.8, 7.0)	5.0 (3.0, 8.0)	
Range	1.0 - 9.0	1.0 - 9.0	
Rate of false positives			0.014†
Mean (SD)	6.0 (2.3)	4.4 (2.5)	
Median (IQR)	6.0 (4.8, 8.0)	4.0 (2.0, 6.0)	
Range	1.0 - 9.0	1.0 - 9.0	
Liability/Medicolegal			0.726†
Mean (SD)	5.6 (1.9)	5.7 (2.6)	
Median (IQR)	5.5 (4.0, 7.0)	6.0 (3.0, 8.0)	
Range	2.0 - 9.0	1.0 - 9.0	
Burden of documentation			0.050†
Mean (SD)	4.3 (2.9)	5.6 (2.4)	
Median (IQR)	3.5 (2.0, 7.0)	5.0 (4.0, 8.0)	
Range	1.0 - 9.0	1.0 - 9.0	
Burden of counseling/integrating into a busy practice			0.021†
Mean (SD)	3.5 (2.1)	5.0 (2.3)	
Median (IQR)	3.5 (2.0, 4.2)	6.0 (3.0, 7.0)	
Range	1.0 - 8.0	1.0 - 9.0	
Patient anxiety for a positive result			0.359†
Mean (SD)	4.0 (2.8)	4.5 (2.3)	
Median (IQR)	3.0 (1.8, 5.5)	4.0 (3.0, 6.0)	
Range	1.0 - 9.0	1.0 - 9.0	
False reassurance with a negative result			0.469†
Mean (SD)	5.7 (3.0)	5.2 (2.6)	
Median (IQR)	7.0 (3.0, 8.2)	5.0 (3.0, 7.0)	
Range	1.0 - 9.0	1.0 - 9.0	

continue...

...Continuation

Reviewing the test	Role/Physician		p value
	Not physician (n=20)	Physician (n=53)	
How would you review a POSITIVE Galleri GRAIL test result with a patient in most cases?, n (%)			0.770*
Patient portal/electronic communication	1 (5)	4 (8)	
Phone call	7 (37)	20 (39)	
In-person visit	8 (42)	23 (45)	
Send to subspecialist for interpretation	3 (16)	4 (8)	
Missing	1	2	
How you review a NEGATIVE Galleri GRAIL with a patient in most cases?, n (%)			0.187*
Patient portal/electronic communication	15 (79)	44 (88)	
Phone call	3 (16)	2 (4)	
In-person visit	0 (0)	3 (6)	
Send to subspecialist for interpretation	1 (5)	1 (2)	
Missing	1	3	
**Department and role**	**Role/Physician**		**p value**
	**Not physician (n=20)**	**Physician (n=53)**	
Please indicate the department in which you primarily see patients, n (%)			
Community Internal Medicine (CIM)	4 (20)	13 (25)	
Family Medicine (FAM)	13 (65)	21 (40)	
General Internal Medicine (GIM; including Executive Health, Development, Consultative Medicine, and International Health)	2 (10)	19 (36)	
None of the above	1 (5)	0 (0)	

*Fisher's Exact Test; †Kruskal-Wallis rank sum test.

SURVEY 1

Familiarity

1. What is your level of familiarity with the GRAIL Galleri® test and/or blood-based multicancer early detection panels (MCEDs) in general?
   a. First time hearing of them
   b. Heard of them but not familiar with what they are
   c. Some degree of familiarity but have not ordered/interpreted
   d. Previously ordered/interpreted these tests
   e. Routine/frequent use in everyday practice

"The GRAIL Galleri® test uses a blood specimen to identify circulating tumor DNA (ctDNA) in a patent's blood. The parent company Grail reports that this test can also identify the likely site of origin if ctDNA is detected (e.g. ovarian versus colon cancer). This test is currently available for ordering with a healthcare provider's prescription/signature.

Whether you are familiar with these tests or not, the subsequent questions will help assess many aspects of these tests in the primary care setting.

The following questions will gather your opinion on multiple aspects of the GRAIL Galleri® MCED test."

1. Have you previously ordered other test(s) based on circulating tumor DNA (ctDNA)?
   a. Yes
   b. No

"Circulating tumor DNA (ctDNA) is DNA released from lysing tumor cells that circulates through a patient's blood stream. It can be detected at very small levels, typically using polymerase chain reaction (PCR) based blood tests.

ctDNA tests have shown promise in diagnosis, assessing response to therapy, predicting recurrence, and other topics in multiple different cancers."

Costs

1. On average, to what extent will health insurance cover the cost of GRAIL Galleri® multicancerl early detection panel?
   a. Fully covered (no cost to patients)
   b. Partially covered (i.e. copay)
   c. Conditionally covered (i.e. certain ages, groups, insurance tiers)
   d. Not covered (100% out-of-pocket to patient)
2. What is your best estimate of the out-of-pocket cost of the GRAIL test to most patients?
   a. $100
   b. $1000
   c. $1500
   d. $10000

"This test costs $979 and is currently a 100% out-of-pocket expense for patients

Click 'Next Page' to proceed through the survey"

1. What percentage of your patients would be interested in undergoing this test at its current price ($949)?
   a. Very few if any (<20%)
   b. Some, but not many (<50%)
   c. Many, but not all (>50%)
   d. All / nearly all (>80%)

"The next set of questions will solicit your opinion on the test characteristics and performance.

Below is a brief background on the test to review before the next set of questions.

'Based on a clinical study of people ages 50 to 79, around 1% received a Cancer Signal Detected result which included predicted Cancer Signal Origin(s). After diagnostic evaluation, around 40% of people received a confirmed cancer diagnosis (Positive Predictive Value of 40%).

In another clinical study that included participants with newly diagnosed cancer, 51.5% of these participants received a Cancer Signal Detected result (sensitivity) and a predicted Cancer Signal Origin across 50 different cancer types. The predicted Cancer Signal Origin (cancer location) was accurate 88.7% of the time. The study also included participants without cancer, 0.5% of these participants received a Cancer Signal Detected result (false positive).'"

Test Information & Characteristics

1. In your opinion, this test is effective at detecting most early-stage cancers
   a. Agree
   b. Disagree
2. This test is an effective replacement for current screening (*e.g*. Pap smear, colonoscopy, mammography, low-dose lung CT, prostate-specific antigen)
   a. Yes
   b. No

"From the GRAIL Galleri® website:

'IMPORTANT SAFETY INFORMATION

The Galleri test is recommended for use in adults with an elevated risk for cancer, such as those aged 50 or older. The Galleri test does not detect all cancers and should be used in addition to routine cancer screening tests recommended by a healthcare provider. Galleri is intended to detect cancer signals and predict where in the body the cancer signal is located. Use of Galleri is not recommended in individuals who are pregnant, 21 years old or younger, or undergoing active cancer treatment.

Results should be interpreted by a healthcare provider in the context of medical history, clinical signs and symptoms. A test result of "No Cancer Signal Detected" does not rule out cancer. A test result of "Cancer Signal Detected" requires confirmatory diagnostic evaluation by medically established procedures (e.g. imaging) to confirm cancer.

If cancer is not confirmed with further testing, it could mean that cancer is not present or testing was insufficient to detect cancer, including due to the cancer being located in a different part of the body. False-positive (a cancer signal detected when cancer is not present) and false-negative (a cancer signal not detected when cancer is present) test results do occur.'"

<u>Ordering</u>

1. Please rank in order (1=most appropriate, 4=least appropriate) whom you think should primarily be ordering this test
   a. Primary care providers (PCPs)
   b. Oncologists
   c. Medical genomics
   d. Subspeciality-specific (i.e. GI providers order to screen for GI cancers, OBGYNs for gynecologic cancers, etc.)
2. For an interested patient with an initially negative MCED result, I would generally order this test again every __.
   a. 1 year (annually)
   b. 5 years
   c. 10 years
   d. One-time only
   e. Not sure
3. I would NOT order this test in a patient younger than __.
   a. <please enter value>
4. I would NOT order this test in a patient older than __.
   a. <please enter value>

<u>Results & Interpretation</u>

1. Would you feel comfortable interpreting a NEGATIVE MCED with a patient.
   a. Yes
   b. No
2. Would you feel comfortable interpreting a POSITIVE MCED with a patient.
   a. Yes
   b. No
3. In your opinion, who should be interpreting the results of a GRAIL Galleri® test with the patient (regardless of who ordered the test)?
   a. Primary care providers
   b. Oncologists
   c. Medical genomics specialists
   d. Subspecialty based on positive signal (*e.g*. GI for GI cancers, OBGYN for gynecologic cancers, urologists for GU cancers)
4. How concerned are you about the amount of time you anticipate spending on counseling patients on whether to undergo the GRAIL Galleri® test?
   a. Not at all
   b. A little
   c. Somewhat
   d. Quite
   e. Very
5. How concerned are you about the amount of time you anticipate spending on interpreting the results of the GRAIL Galleri® tests and communicating results to patients?
   a. Not at all
   b. A little
   c. Somewhat
   d. Quite
   e. Very
6. A semi-automated electronic medical record feature (*e.g*. Epic smartphrase) would be sufficient medicolegal documentation for a POSITIVE result.
   a. Agree
   b. Disagree

7. A semi-automated electronic medical record feature (*e.g*. Epic smartphrase) would be sufficient medicolegal documentation for a NEGATIVE result.
   a. Agree
   b. Disagree

Downstream Evaluation

1. For a positive MCED result, what would be your next step?
   a. Order disease-directed evaluation (*e.g*. EGD for esophageal signal)
   b. Refer for oncology and/or medical genomics consultation and defer further testing to the subspeciality consultant
   c. Refer to oncology and/or medical genomics and concurrently order disease-directed evaluation
2. How comfortable would you feel with ordering disease-directed subsequent testing for a positive GRAIL Galleri® MCED test?
   a. Not at all
   b. Comfortable for a limited subset of cancer signals (*e.g*. mammogram for breast signal, colonoscopy for colorectal signal)
   c. Comfortable doing this with most/all cancer signals

Counseling

1. Please rank your concerns regarding the GRAIL Galleri® MCED test (1=Biggest concern, 9=Least concern)
   a. Cost to patient
   b. Cost to healthcare system (downstream testing, subspecialty referral)
   c. Impact on health equity (*i.e*. access to a $979 test)
   d. Rate of false positives
   e. Liability
   f. Burden of documentation
   g. Burden of counseling / integrating into a busy practice
   h. Patient anxiety for a positive result
   i. False reassurance with a negative test
2. How would you review a POSITIVE GRAIL Galleri® test result with a patient in most cases?
   a. Patient portal
   b. Phone call
   c. In-person visit
   d. Prefer to send to subspecialist for interpretation
3. How would you review a NEGATIVE GRAIL Galleri® test result with a patient in most cases?
   a. Patient portal
   b. Phone call
   c. In-person visit
   d. Prefer to send to subspecialist for interpretation

Background/Demographic

1. Please indicate the department in which you primarily see patients.
   a. Community Internal Medicine (CIM)
   b. Medallion (MDL)
   c. General Internal Medicine (GIM; including Executive Health, Development, Consultative Medicine, International Health)
   d. Family Medicine (FAM)
   e. Women's Health Internal Medicine (WHIM)
2. Please indicate your level of training
   a. Physician assistant
   b. Nurse practitioner
   c. Physician
   d. None of the above (survey will terminate)
